# Esthetic outcome of implant-based reconstructions in augmented bone: comparison of autologous and allogeneic bone block grafting with the pink esthetic score (PES)

**DOI:** 10.1186/1746-160X-10-21

**Published:** 2014-05-28

**Authors:** Markus Schlee, Jan-Friedrich Dehner, Katja Baukloh, Arndt Happe, Oliver Seitz, Robert Sader

**Affiliations:** 1 Private practice for implantology and periodontology, Forchheim, Germany, Johan Wolfgang Goethe University, Bayreuther Strasse 39, 91301 Frankfurt, Germany; 2 Private practice, Rome, Italy and Eastman Dental Hospital, Rome, Italy; 3 Private practice, Oberursel, Germany; 4 Private practice for implantology and oral surgery, Münster, Germany; 5 Department for Oral and Maxillofacial Surgery, Johann Wolfgang Goethe-University, Frankfurt, Germany

**Keywords:** Autogenous bone block, Allograft bone block, Pink esthetic score, Long term result

## Abstract

**Introduction:**

To determine the esthetic outcome of implant-based reconstructions after autologous and allogeneic bone grafting.

**Methods:**

From 2003 to 2009, 67 patients underwent alveolar ridge augmentation and were enrolled in the study, 41 meet the inclusion criteria and 31 agreed to take part in the study. Patients were 18-69 years old (mean: 49.3 ± 13.8 years), and predominantly female. Patients received bone block grafts either autologous (n = 48) (AUBB) or allografts (ABB) (n = 19). Implants were inserted 4-7 months (autografts) or 5-6 months (allografts) after bone grafting. The Pink Esthetic Score (PES) as well as radiographic and subjective assessments were employed for the outcome analysis. The PES was assessed twice within one month based on digital photographic images that were randomly rearranged between evaluations by three independent, experienced investigators.

**Results:**

Across all observations and investigators, the average PES was 7.5 ± 2.6 without differences between implants inserted in auto- and allografted bone, respectively. Patients assessed the allograft procedures as less painful and would have repeated it more often. The intra-rater reliability was excellent (correlation coefficients 0.7-0.9). The inter-observer agreement was lower (correlation coefficients 0.6-0.8).

**Conclusions:**

Bone grafting with ABB allografts yields equivalent results to autologous grafting, and patients appreciate the omission of bone harvesting. The PES is a reliable method but should be performed by the same individual.

## Introduction

Dental implants in augmented bone have evolved into an established treatment modality with functional results practically on par with implants in native bone [1-4]. This modality is of a particular potential value for the integration of fixed dentures in edentulous and/or atrophic jaws where it may provide a growth impulse by conducting bite forces into bone and thus terminate or reverse alveolar bone resorption [5-8]. However, the evidence for the ’real world’ practical value of implant-based fixed dentures in terms of long-term functional and esthetic survival in the rehabilitation of edentulous jaws is presently limited [9].

Implantation strictly in native bone requires a certain minimum bone volume and therefore significantly restricts the number of eligible patients. Due to very diverse criteria for decision-making with respect to bone augmentation in general and the specific method in particular, it is impossible to assess or even roughly estimate the number of patients eligible for implantation in native vs. augmented bone. This insecurity notwithstanding, it can be assumed that the availability of techniques for the reconstruction and regeneration of lost hard and soft tissues has exponentially increased the number of candidates for implant-based reconstructions and dentures. Moreover, bone and soft tissue provide the implantologist with extended opportunities for implant placement and therefore crucially facilitate a three-dimensional implant placement that is optimal both in terms of function and esthetics [10].

One of the key success criteria is the achievement and maintenance of a sufficient bone height to support the implant mechanically and provide a sound basis for soft tissues for sustained esthetically pleasing results. A positive correlation between bone height and soft tissue margin are well accepted [11-14]. From a reconstructive point of view, autologous bone is doubtlessly the ideal material for augmentation [6], and a substantial body of evidence strongly suggests its suitability e. g., [1-3,15,16]; therefore, alternative materials have to be assessed in comparison to autologous bone as the currently most established standard [2,4,17].

However, autologous bone transplantation has one key disadvantage: The bone is harvested from either intra- or extraoral sites, both of which have particular shortcomings. Bone harvesting from the mandibular ramus – the most frequently tapped intraoral bone reservoir – generally shows a low morbidity, but provides a limited bone volume; harvesting from the chin or peripheral sites such as tibia and iliac crest yields more than sufficient amounts of bone but are burdened with a considerable morbidity, e. g. persistent pain and sensory defects [16,18-20]. Therefore, donor site considerations command caution in treatment recommendation, and a material that is not autologous but equivalent in implant integration suitability is obviously of interest and thus subject of prolific research.

Published studies suggest that allogeneic bone grafts may provide such an alternative [15,20-23], but the available evidence currently does not sufficiently support the decision for autologous or allogeneic bone grafting, respectively [24], requiring further studies and in particular comparative trials.

Recently, it is increasingly understood that ‘implant stable *in situ*’ is a necessary but not sufficient precaution for treatment success; in fact, in addition to a fulfillment of the desired function, a flawless appearance is paramount at least when the implant is located in the esthetic zone.

From the patients’ perspective, the appearance of peri-implant soft tissue and the prosthetic superstructure presents a very important criterion for the success of the implant treatment. In 2003, Vermylen et al. published a study on patient satisfaction with single-tooth implant restorations and stressed that an esthetically satisfactory result from this kind of treatment was a main concern for patients [25].

Belser et al. [26] criticized the disregard in assessing the appearance of implant-prosthetic restorations in connection with clinical studies. Based on a review of the success of single-tooth implant restorations in the anterior maxilla they came to the conclusion that ‘the aesthetic result in scientific studies is generally poorly documented and presents no success criteria.’ The authors proposed an esthetic score in order to obtain objective results regarding the esthetic outcome of implant treatments.

The ‘pink’ (i. e., soft-tissue) esthetics of an implant-based restoration is not only a result of the surgical management of the soft tissues during implantation and denture insertion but also a function of the dimensional stability of the bone structures beneath. A satisfactory early result can be seriously marred by bone resorption and subsequent apical relocation of soft tissue margins in the long term.

The present study investigates the respective esthetic outcome of implants inserted in bone augmented with autologous and allogeneic grafts, respectively, employing the Pink Esthetic Score (PES), an established evaluation instrument for bone resorption.

## Materials and methods

### Patients

Between Jan 1^st^, 2003 and Dec 31^st^, 2009, a total of 67 patients (48 with AUBB and 19 with ABB) underwent alveolar ridge augmentation and implant insertion in the private practice of one of the authors (M.S.). In order to be eligible for the present study, patients had to fulfill the following criteria:

• Inclusion criteria

○ ASA physical status class 1

○ Alveolar ridge augmentation and implant insertion within the observation period

○ Integration of fixed dentures

○ Bone augmentation with autologous bone or Puros® allografts (Tutogen Medical, Neunkirchen, Germany)

○ Legal and cognitive fitness for informed consent

• Exclusion criteria

○ ASA physical status class 2-6, systemic disease

○ Pregnancy

According to these criteria, 41 patients were eligible and received a written invitation for a re-examination in February 2010, and 31 of these patients (75.6%) agreed to undergo the clinical, photographical and radiological evaluation after meticulous information about possible hazards and benefits. The study complied with the declaration of Helsinki and pertinent Good Clinical Practice guidelines.

Patients were 18-69 years of age (mean: 49.3 ± 13.8 years), and predominantly female. Only two subjects were current smokers, and one patient was a former smoker (Table 1).

**Table 1:** Demographic properties of the sample and baseline data

**Criterion**	**Total**	**Autograft**	**Allograft**
Number of patients (n, %)	31	21 (67.7%)	10 (32.3%)
Males/females (n, %)	8/23 (25.8/74.2%)	4/17 (19.0/81.0%)	4/6 (40.0/60.0%)
Age (years)^‡^	18-69 (49.3 ± 13.8)	18-69 (49.0 ± 15.6)	37-64 (49.8 ± 9.7)
Smoking status (n, %)			
Non-smoker	28 (90.3%)	18 (85.7%)	-
Ex-smoker	1 (3.2%)	1 (4.8%)	10 (100.0%)
Smoker (≤10 cig./day)	2 (6.5%)	2 (9.5%)	-
Treatment reason (n, %)			
Ridge atrophy	17 (54.8%)	11 (52.4%)	6 (60.0%)
Endodontal	6 (19.4%)	4 (19.0%)	2 (20.0%)
Periodontal	5 (16.1%)	3 (14.3%)	2 (20.0%)
Failed implant	2 (6.5%)	2 (9.5%)	-
Gap widening	1 (3.2%)	1 (4.8%)	-
Number of implants (n, %)	48	33 (68.8%)	15 (31.2%)
Time since augmentation (months)^‡^	11-88 (49.5 ± 25.8)	21-88 (59.5 ± 23.7)	11-53 (28.6 ± 15.8)

‡ range (mean ± std. deviation).

The most frequent indication for implant insertion in augmented bone was alveolar ridge atrophy after extraction (n = 17, 54.8%), followed by endodontal (n = 6, 19.4%) and periodontal destruction (n = 5, 16.1%), replacement of failed implants (n = 2, 6.5%) and gap widening after orthodontic therapy for aplasia (n = 1, 3.2%).

There were no significant group differences with respect to demographics and baseline data (Table 1).

### Surgical and restorative methods

All procedures were performed by the same dental surgeon (M.S.) under local anesthesia. After a meticulous examination, all patients received careful oral hygiene instructions and a professional tooth cleaning before the actual surgery. Ridge augmentation was considered necessary when the desired implant diameter plus 3 mm of bone width was not present.

After the allograft material was introduced (December 2005), the augmentation method was chosen according to the patient’s preference after a mutual consideration of the respective advantages and drawbacks of either method with the dental surgeon.

**Figure 1:**
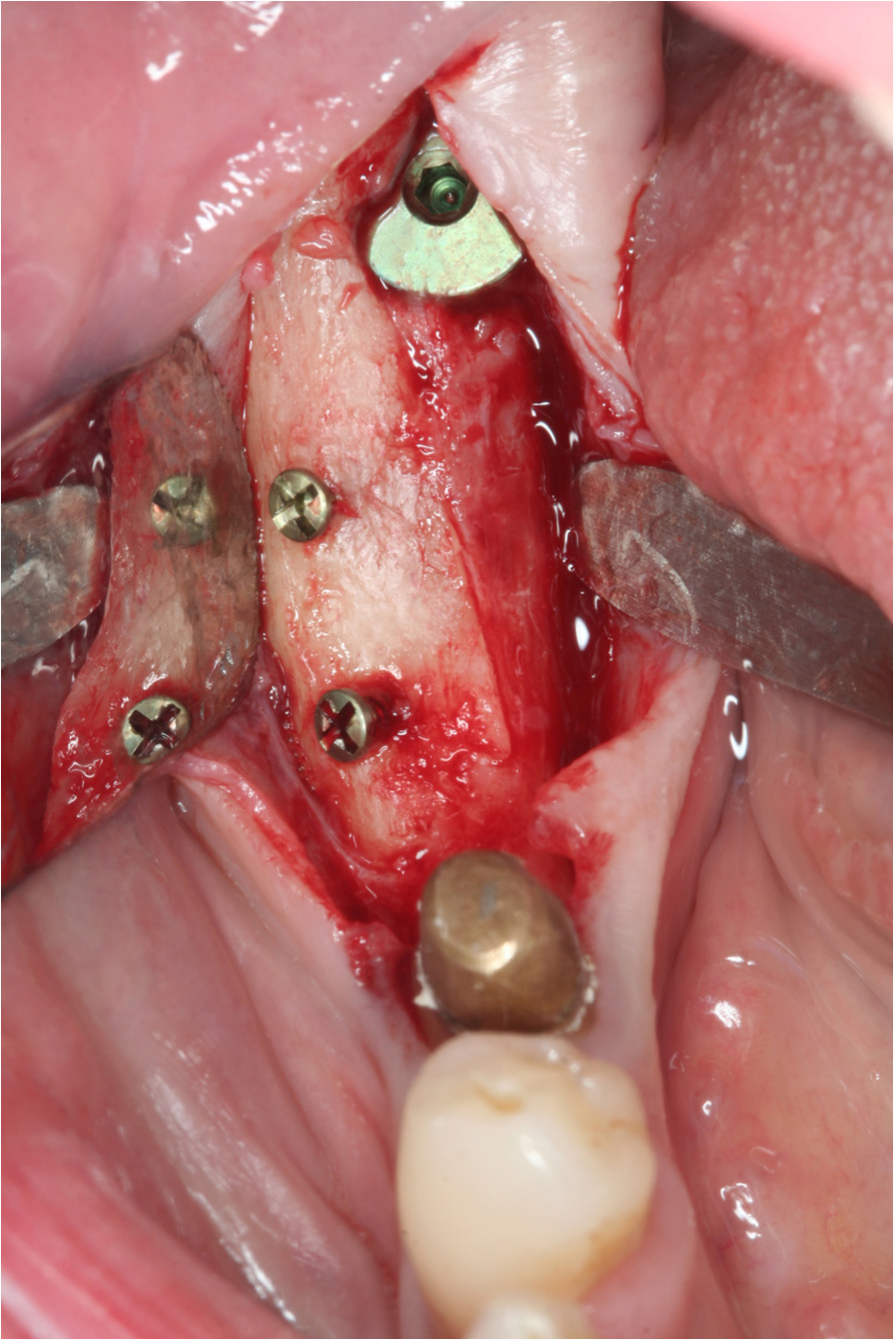
**Well integrated autograft harvested from the ramus area.** Bone blocks from linea obliqua externa are consisting mainly of cancellous bone. Even after 6 months the block seems to be integrated very nicely but still has a white color indicating incomplete turn over in living bone.

**Figure 2:**
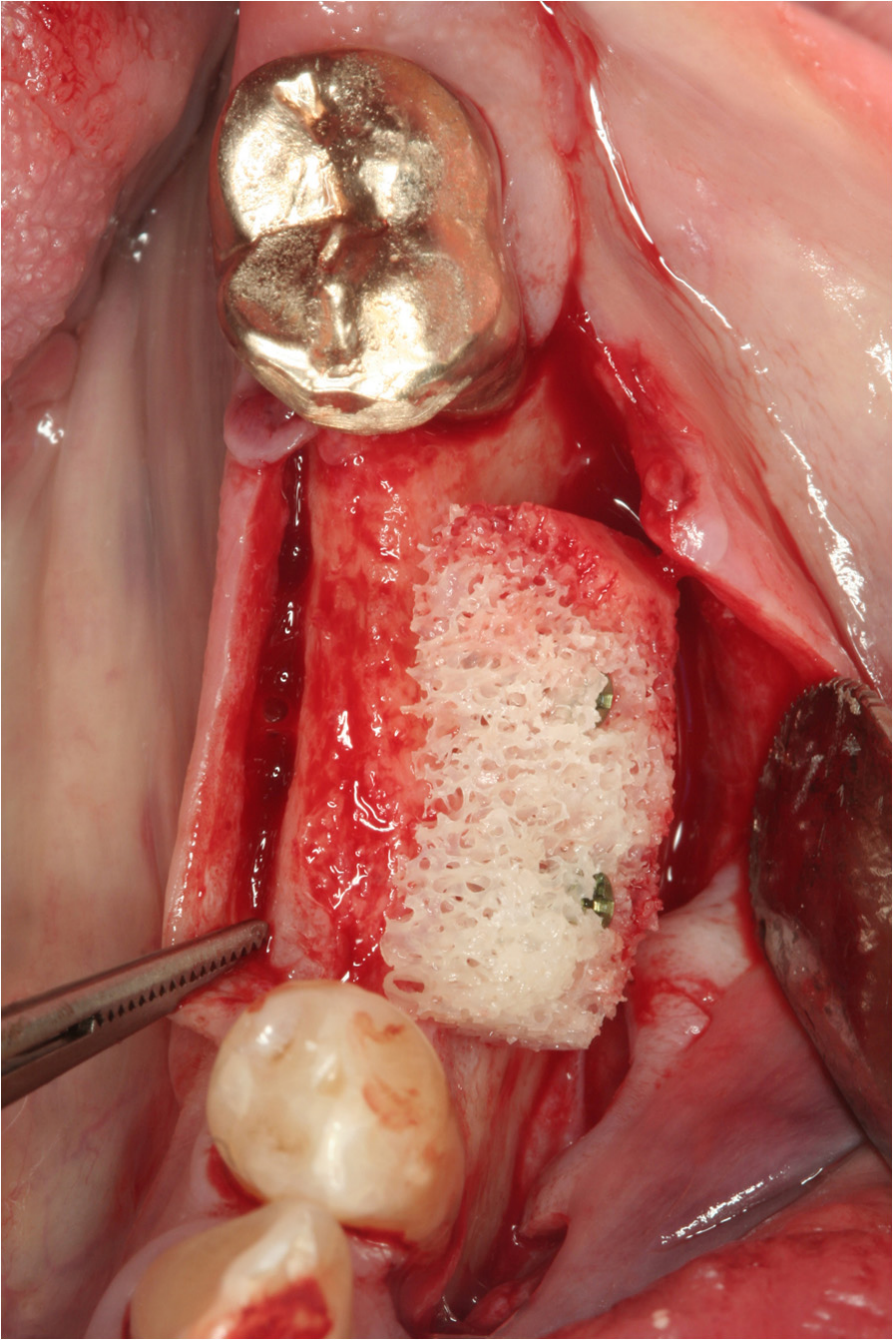
**ABB reduce surgery time and trauma.** At baseline the allograft made of spongous bone derived from femur heads of living donators is adapted to the recipient site and fixed by screws.

**Figure 3:**
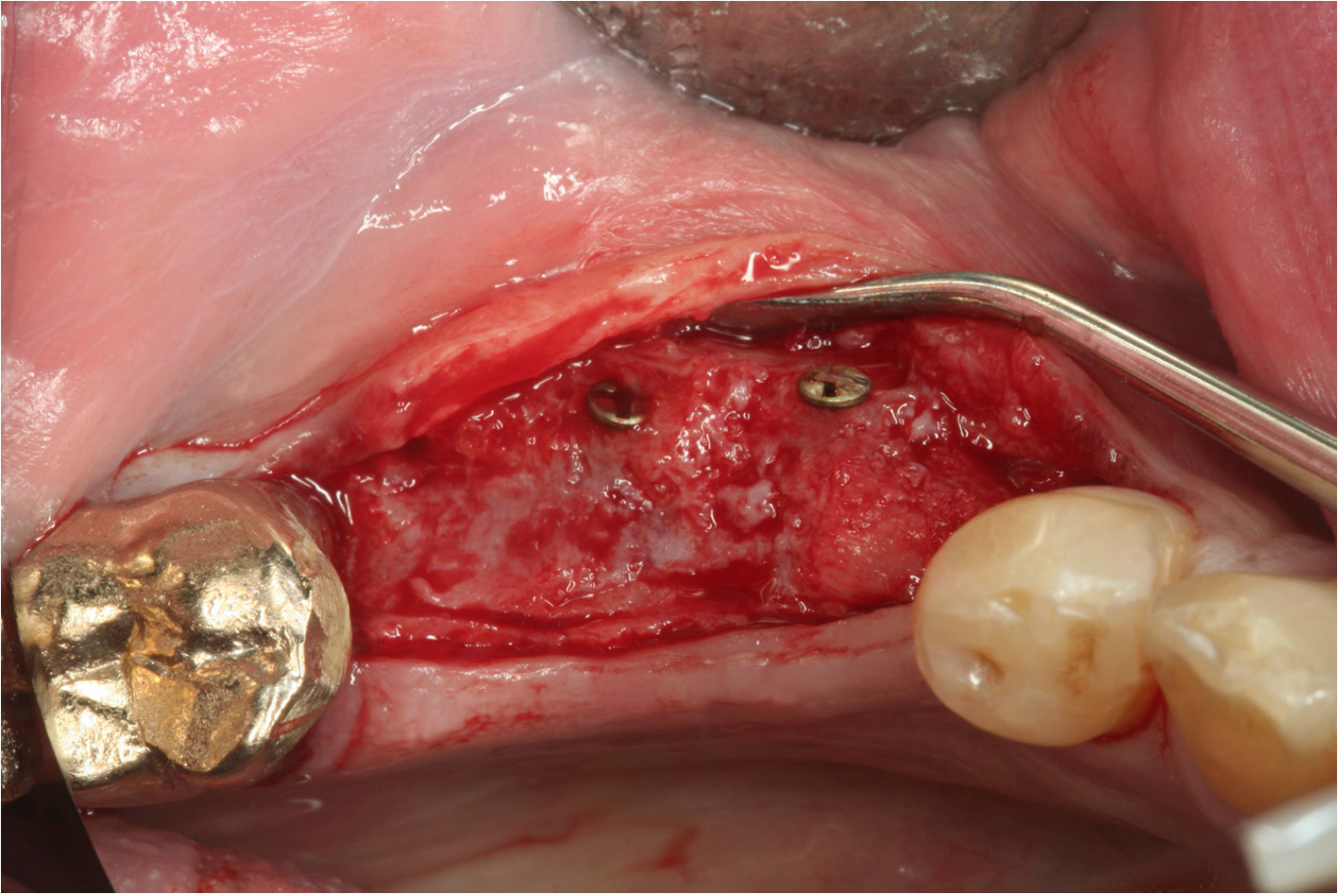
**ABB integrated nicely after 6 months.** Clinically the ABB seems to be integrated more nicely than the autograft. It is bleeding and the former spongous structure changed and a closed surface is visible.

In patients receiving autografts, bone material was harvested from the *linea obliqua externa from ramus* (Figure 1) in all but one patient, in whom bone from the iliac crest was used. The ABB allografts (Puros®, Tutogen Medical, Neunkirchen, Germany) used in the study are manufactured from human tibia or femur head spongiosa which was harvested after joint replacement rom living donators and processed with a proprietary method that chemically preserves the collagen matrix and tissue integrity while removing unwanted materials such as cells, fat, antigens and viruses, and inactivating pathogens (Tutoplast® method) (Figures 2 and 3).

After crestal incision, lingual and buccal full thickness flaps were raised and the latter extended past the mucogingival junction with a split thickness technique. The cortical bone of the recipient area was perforated with a drill to improve transplant integration. Bone blocks were shaped at chairside and fixed with osteosynthesis screws to avoid micromobility. Remaining incongruities were filled with smaller bone chips or – in two cases – BioOss® (Geistlich, Wolhusen, Switzerland), respectively. In all but one patient, the bone block was covered with a membrane (Bio-Gide® [Geistlich, Wolhusen, Switzerland] or Tutodent® [Tutogen Medical, Neunkirchen, Germany]). The flap was closed over the membrane with sutures.

Implants were inserted 4-7 months (autografts) or 5-6 months (allografts) after bone grafting. All implants had an internal connection, were fabricated from titanium grade 4, had sand-blasted, large grid and acid-etched surface (SLA) and were restored with titanium abutments. All the inserted crowns were cemented with Durelon® [Espe, Seefeld, Germany].

### Evaluation methods

The 31 patients received a total of 48 implants (up to four per patient), and the implants were *in situ* for an average of approximately 50 months at re-evaluation. The observation period of autografts was approximately twice as long as for allografts because the latter were only used from December 2005 onwards (Table 1).

Upon re-examination, all implants were fitted with final fixed restorations for at least 6 months.

After accepting the written invitation, patients received an appointment for the examination that took place between March and August 2010. The evaluation included a clinical examination, photographic documentation, ‘right-angle’ radiography and a questionnaire regarding patient satisfaction. Since the objective was peri-implant tissue (and not prosthodontic quality) assessment, the Pink Esthetic Score [27,28] (PES) was chosen as the appropriate, established method for the esthetic evaluation.

For the PES assessment, frontal photographs were taken with a flash strobe with a digital camera mounted on a tripod (Canon 350d, 3456×2304 pixel resolution). When photographs of incisors were taken, the image section was chosen to include the opposite quadrant for comparison. The photographs were composed into a PowerPoint presentation in random order. Three independent, experienced investigators familiar with the PES scoring method who were blinded with respect to the augmentation method and had no other connection to the study reviewed all images on the same portable computer under identical lighting conditions and assigned a PES at their discretion. The score is computed by addition of the point score (from 0=‘very bad’ to 2=‘excellent’) for the seven items mesial papilla, distal papilla, soft-tissue level, soft-tissue contour, alveolar process deficiency, soft-tissue color and texture for a maximum score of 14.

After one month, the three investigators reassessed all images that had been rearranged in a different random order.

Radiographs taken by the ‘right-angle’ technique were routinely taken after implant insertion and upon reexamination. Bone loss was estimated from the difference of both images based on the known implant size by rule of proportion.

Patients rated their own satisfaction with the treatment outcome according to the following questions:

1. Are you satisfied with the esthetic result of the procedure? (Scale from 1=‘very satisfied’ to 5=‘not satisfied at all’)

2. Are you satisfied with the color of the mucosa? (Scale from 1=‘very satisfied’ to 5=‘not satisfied at all’)

3. Would you undergo the same procedure again? (Scale from 1=‘certainly’ to 5=‘certainly not’)

4. On a scale from 1 (‘no pain at all’) to 10 (‘unbearable pain’), how would you rate the pain during the procedure?

5. Do you feel that the esthetic appearance of the restoration kept changing after integration? (Scale from 1=‘very much improved’ to 5=‘very much deteriorated’)

### Statistical analysis

Data were statistically analyzed with the STATISTICA software package (Statsoft, Tulsa, OK) using non-parametric methods with p < 0.05 as the threshold for statistical significance. Inter- and intra-rater reliability were tested with inter-rater correlation coefficients (Spearman’s R).

## Results

### Outcome description

Postoperative complications were rare. All in all, 41 implants healed free of complications. Deep infections that required surgical revision occurred in 3 cases (all after autografts), and in 4 cases (2 of either group) a denudation of the augmented bone required secondary measures. No implants were lost during the observation period.

Across all observations and investigators, the average PES was 7.5 ± 2.6, and the esthetic outcome of implants inserted in auto- or allografted bone, respectively, was virtually identical. The same applied to the amount of bone resorption: The average bone loss was 1.60 ± 1.24 mm in the entire sample and exactly identical after insertion in autograft (1.60 ± 1.37 mm) and allograft (1.60 ± 1.03 mm), respectively. There was no statistically significant correlation between bone loss and observation period in either group (Spearman’s R -0.040 [all implants], -0.186 [autograft] and 0.133 [allograft], p > 0.05 for all) and only a non-significant trend towards lower PES-scores with increasing bone loss (Spearman’s R 0.142, p > 0.05).

There was a substantial and significant influence of number of adjacent implants on the esthetic outcome: Whereas the mean PES was almost 9 points at single-tooth implants, it was on average 2 and 2.5 points lower at restorations on 2-3 and ≥4 implants, respectively. The difference between single-tooth restorations and both other groups was statistically significant (p < 0.05), while the difference between the two latter groups was not. There was no difference between these groups in terms of bone loss (Table 2).

**Table 2:** Outcome in single tooth vs. multiple implants

**Type of restoration**	**Number of implants in group**	**PES (average across all observations)**	**Bone loss (mm)**
Single tooth	18 (37.5%)	8.8 ± 2.3	1.91 ± 1.33
Two or three adjacent implants	23 (47.9%)	6.9 ± 2.5	1.45 ± 1.15
Four or more adjacent implants	7 (14.6%)	6.3 ± 2.4	1.05 ± 1.38
p-value		0.024 (all 3 groups)	>0.05
		0.018 (single vs. 2-3)	
		0.026 (single vs. ≥4)	
		0.561 (2-3 vs. ≥4)	

In contrast to the PES, the subjective assessment by the patients showed some considerable and statistically significant advantages of allograft procedures (Table 3). While patients from both groups rated the esthetic outcome as a whole and the mucosal color practically identical (thus corroborating the PES results), they assessed the autograft procedure significantly more painful (pain score 4.2 ± 2.3 as opposed to 2.5 ± 1.9, p < 0,05), and consequently were more reluctant to undergo the same procedure again, given the same initial position, albeit on a very high level of willingness (1.52 ± 0.67 after autograft, all patients rated ‘1’ after allograft, p < 0.01).

**Table 3:** Subjective outcome assessment

**Criterion**	**Total**	**Autograft**	**Allograft**	**p-value**
Satisfaction with the esthetic result	1-2	1-2	1-2	0.219
	(1.17 ± 0.38)	(1.21 ± 0.42)	(1.07 ± 0.26)	
Satisfaction with the color of the mucosa	1-4	1-4	1-2	0.975
	(1.40 ± 0.61)	(1.39 ± 0.66)	(1.40 ± 0.51)	
Willingness to repeat the procedure	1-4	1-4	1-1	0.005
	(1.35 ± 0.60)	(1.52 ± 0.67)	(1.00 ± 0.00)	
Pain during procedure	1.0-8.5	1.0-8.5	1.0-8.0	0.013
	(3.7 ± 2.3)	(4.2 ± 2.3)	(2.5 ± 1.9)	
Appearance change after restoration	1-4	1-4	1-3	0.048
	(2.40 ± 0.94)	(2.58 ± 0.87)	(2.00 ± 1.00)	

**Figure 4:**
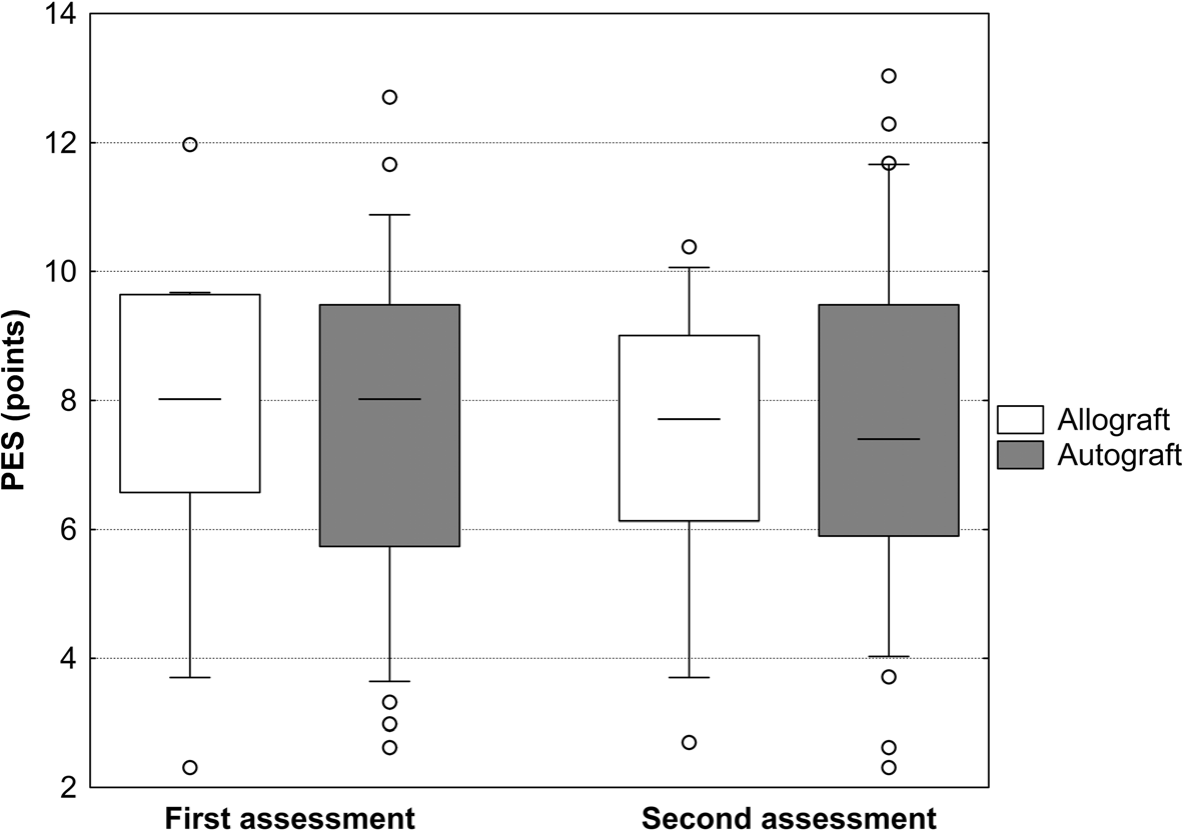
**Pink esthetic score depending on the time of assessment and grafting method.** Three due to the augmentation method blinded investigators performed the PES assessment. After one month, the investigators reassessed all images that had been rearranged in a different random order. There was no appreciable difference between the first and second assessment in either group.

Interestingly, the three implants placed in the first period of allograft procedures showed a somewhat poorer esthetic outcome, while in later years the results were identical with those of autografts (Table 4).The intra-rater reliability was excellent (correlation coefficients between 0.683 [observer 3] and 0.890 [observer 2]), and there was no appreciable difference between the first and second assessment in either group (Figure 4).

**Table 4:** PES values depending on the time elapsed since surgery (number of implants, range [mean ± std. deviation])

**Time between augmentation and examination**	**Total**	**Autograft**	**Allograft**
All implants^¥^	n = 48	n = 33	n = 15
	2.5-12.8 (7.5 ± 2.6)	2.5-10.5 (7.5 ± 2.4)	2.5-12.8 (7.5 ± 2.6)
≤2 years	n = 8	n = 4	n = 4
	5.7-12.8 (8.1 ± 2.4)	5.7-12.8 (8.2 ± 3.2)	6.2-9.7 (8.0 ± 1.7)
>2 - ≤4 years	n = 13	n = 5	n = 8
	3.7-12.2 (8.4 ± 2.1)	6.5-12.2 (8.6 ± 2.2)	3.7-10.5 (8.3 ± 2.2)
>4 - ≤6 years	n = 11	n = 8	n = 3
	2.5-9.5 (6.5 ± 9.3)	4.0-9.5 (7.2 ± 2.0)	2.5-7.3 (4.8 ± 2.4)
>6 years	n = 16	n = 16	n = 0
	2.5-12.0 (7.2 ± 3.0)	2.5-12.0 (7.2 ± 3.0)	

¥ Average of all assessments (three investigators and two evaluations).

**Figure 5:**
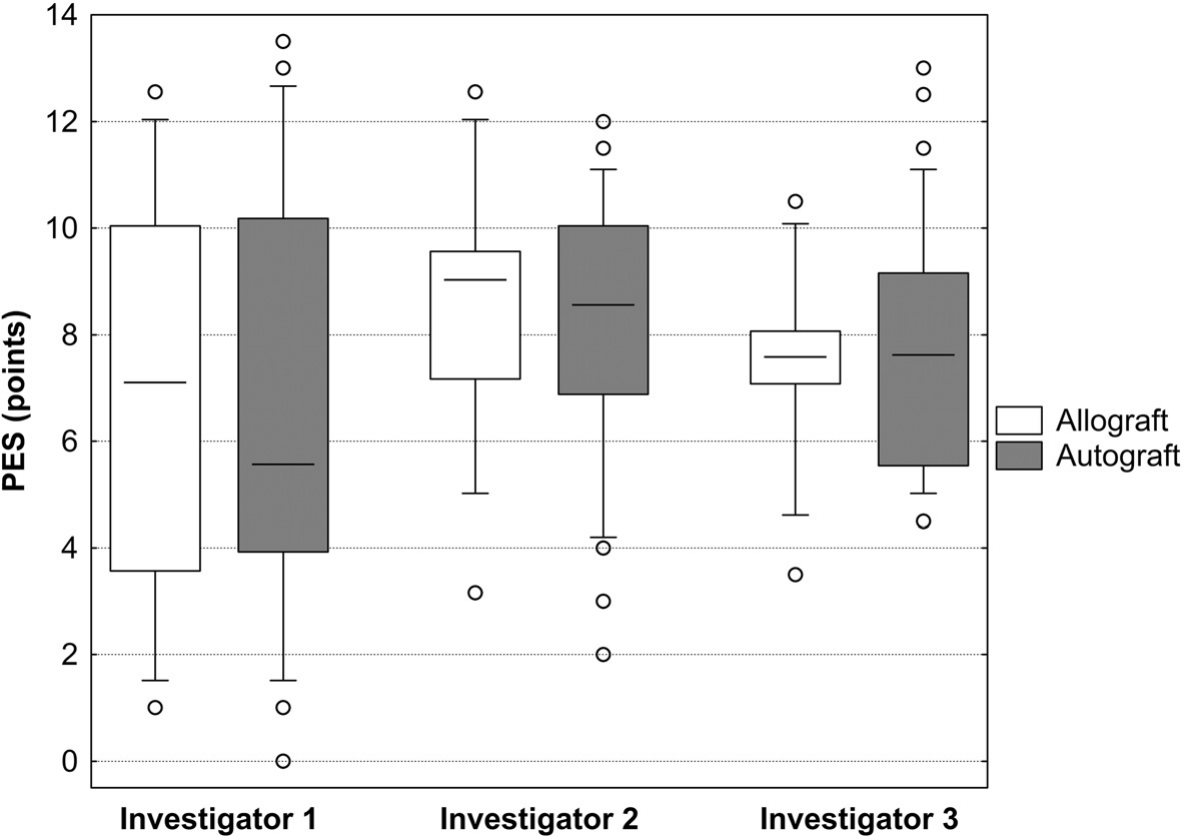
**Pink esthetic score depending on the investigator and grafting method**. The intra-rater reliability between the three investigators was excellent.

**Figure 6:**
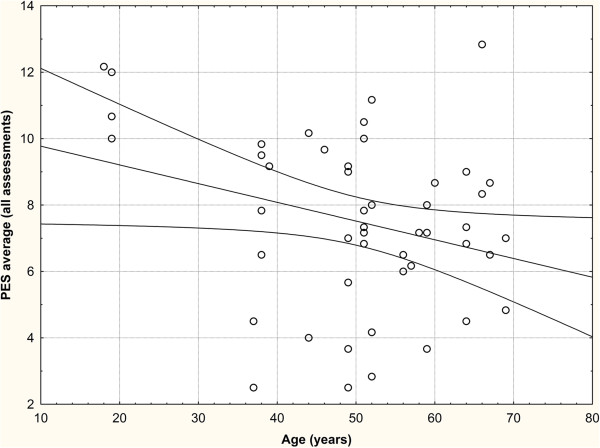
**Correlation between patient age and PES (p < 0.05).** Age had significant influence on the PES. The 95% confidence interval for patients in their 20s roughly reached from 8 to 11 points, while it was between 4 and 8 in patients in their 80s.

However, there were considerable inter-rater differences with approximately 1-2 point-increments (Figure 5): The Investigator 2 (8.4 ± 2.5 points overall average) consistently rated the pink esthetics consistently higher than Investigator 3 (7.5 ± 2.2, p = 0.003) and especially Investigator 1 (6.6 ± 3.9, p = 0.0002). The difference between the latter two was also statistically significant (p = 0.021). The inter-rater reliability of the PES in the present study was somewhat lower than the intra-observer agreement, Spearman’s R ranging between 0.58 and 0.66 (Table 5).Once again, the grafting material played no role with respect to the esthetic outcome assessed by either investigator (Figure 5).The only confounder with a statistically significant influence on the esthetic outcome was age: The 95% confidence interval for patients in their 20s roughly reached from 8 to 11 points, while it was between 4 and 8 in patients in their 80s (Figure 6).

**Table 5:** Inter-observer agreement for PES assessment at both evaluations

**Comparison**	**Time point**	**Spearman’s R**	**p-value**
Observer 1 vs. observer 2	T1	0,575806	0,000019
Observer 1 vs. observer 3	T1	0,665267	0,000000
Observer 2 vs. observer 3	T1	0,590510	0,000010
Observer 1 vs. observer 2	T2	0,588966	0,000011
Observer 1 vs. observer 3	T2	0,649044	0,000001
Observer 2 vs. observer 3	T2	0,595960	0,000008

## Discussion

Using the pink esthetic score (PES) changes in the esthetic outcome over time and also between two or more cohorts can be evaluated. Despite its non-randomized design and limited sample size, the present trial yields two rather interesting results pertinent to clinical and methodological considerations, respectively. With regard to the main study issue – the respective esthetic outcome quality of allograft- in comparison to autograft-based implants – the results convincingly point at an absence of appreciable differences. There were no differences in PES between autografts and allografts so ever, and this observation was robust across examinations and observers. The result is corroborated by an equally conspicuous lack of differences in the subjective assessment of esthetic outcome by the patients and by the absence of differences in bone loss between groups. The issue of a possibly underpowered sample size with a resulting type-2-error (false negative result of statistical testing) is pointless due to the complete absence of any appreciable group differences favoring autografts.

Although the trial was mainly designed to demonstrate the absence of inferiority of allografts as compared to autografts, it emphasizes the advantage of foreign materials by means of a significantly reduced pain perception and a more marked willingness to repeat the procedure in patients receiving allografts. Whereas the latter result might be biased by the fact that all patients with allografts, but only about half of the patients with autografts (those with procedures after the allograft introduction) had chosen the treatment method themselves, this is hardly the case with respect to esthetic outcome and bone loss; all in all, the non-inferiority of allograft-based implant insertion can be concluded safely and convincingly, and the possible advantages of allografts are subject to the patients’ choice anyway.

The potential benefit of the ABB allografts employed in the present study is emphasized when the time course is considered: The first three allograft procedures yielded comparably poor esthetic results (PES average 4.8 points) whereas subsequent procedures consistently showed PES scores ≥8 points. This indicates a learning curve in early allograft application that was by the way also apparent in autografts, although to a lesser extent (see Table 3).

Single tooth implants had a higher PES score than multiple implants but the same bone loss (see Table 2). The reason for this can only be speculated on. Probably it is the fact that the periodontium of neighboring teeth maintains the bone beside the teeth and by that also the soft tissue which causes higher PES scores.

Subjectively, the patients assessed their respective esthetic outcome almost uniformly as excellent. The fact that patients rated the ‘appearance change after the procedure’ significantly better after allografts in the absence of pertinent differences in individual esthetic outcome criteria demonstrates the profound influence of overall satisfaction that includes the discomfort experienced during and after surgery. Therefore, despite the relative rarity of serious donor site complications [16,18], the additional surgical procedure during bone harvesting appears to be a significant strain for the patients.

The second main result of the present trial pertains to the methodology of PES evaluation: Whereas the reproducibility of results in individual observers was extremely high, inter-rater reliability was rather modest at first glance. However, when considering the technical aspects of the PES determination – the PES consists of 7 variables (scored from 0-2) all of which are prone to some subjectivity –, this is hardly surprising, and published data are in agreement with correlation coefficients of 0.6-0.8 [28,29] and substantial inter-observer differences [27,28]. The available evidence strongly suggests a tight correlation between PES outcome and patients’ subjective assessment [10,30], and this strongly underscores the PES’s value as an outcome assessment instrument. The excellent intra-observer agreement demonstrated in the present trial and the literature [14,27-29] corroborates its value for longitudinal observations that are crucial in study settings as well as in quality-controlled treatment environments. However, our results strongly suggest that, if and whenever possible, PES assessments should be performed by the same individual to assure a high reliability of results.

Overall, the PES scores in the present study are somewhat lower than in other trials, for instance Fürhauser’s original publication [27]; while Fürhauser – as well as Gehrke et al. [28] – reported an average of around 9 points, the present study showed a gross average of 7.5 points. However, this does not necessarily indicate an objectively inferior outcome: Fürhauser himself as well as other investigators [28,29] described substantial inter-observer differences especially when different groups of professionals where compared. It appears plausible that observers who are regularly involved in treatment and assessment of patients with augmentations may be more critical than professionals who encounter those tasks more occasionally. Moreover, there are several published trials with PES outcome on par with those of the present trial [29,31].

In support of our first main result – the absence of an esthetical penalty when employing ABB allografts – the literature mainly shows equivalent results of autografts and allografts in general and the Puros® ABB allografts in particular. A recently published histovolumetric study in 30 patients by Schmitt et al. [32] demonstrated that Puros® allografts were virtually equivalent to autologous transplants in terms of newly formed bone, and markedly superior to other substitute materials (i. e., biphasic calcium phosphate and anorganic bovine bone), thus confirming previously published results [33-35]. No similarly large series of Puros® allografts in lateral ridge augmentation has been published so far, but a report on three cases [36] provides provisional corroboration. In confirmation of extensive reviews [3,15,37], the success of augmentation procedures appears to be relatively independent of the material employed.

According to our own trial as well as several clinical studies [36,38], Puros® ABB allografts facilitate the successful implant integration in severely bone-deficient sites with excellent functional and esthetic results; their key advantage over autologous transplantation is the omission of bone harvesting and over other allograft procedures apparent advantages in terms of new bone formation.

Therefore, implant-based constructions inserted in bone after augmentation with ABB allografts appear to be a viable alternative to autograft or other allograft procedures. However, the best available method ought to be chosen individually after careful and comprehensive consideration of all patient- and procedure-related advantages and disadvantages [7,39]. For the time being, autologous bone transplantation remains the ‘gold standard’ [6,40], but this may have to be reconsidered when more reliable evidence about the outcome of allograft procedures becomes available [3,9,41].

## Conclusion

Autologous bone grafts are assumed to be the gold standard in augmentation procedures but allografts are used widely as an alternative to avoid second site surgery. No study compared the outcome of both methods yet. This retrospective study analyzed the long term results of AUBB and ABB was 49.5 ± 25.8 months treated by one surgeon. The mean observation period, no implant was lost. There was no statistical significant difference in bone loss in both groups. The average PES was 7.5 ± 2.6 without differences between implants inserted in auto- and allografted bone, respectively. Patients assessed the allograft procedures as less painful and would have repeated it more often.

Bone grafting with ABB allografts yields equivalent results to AUBB grafting, and patients appreciate the omission of bone harvesting. The PES is a reliable method to assess differences.

## Informed consent

Written informed consent was obtained from the patient for the publication of this report and any accompanying images.

## Competing interest

The authors have no conflicts of interest to declare.

## Authors’ contributions

MS: Concept and design, Data analysis and interpretation, Drafting article, Critical revision of article, Approval of article, Data collection. J-FD: Data interpretation, Drafting article, Critical revision of article, Statistics. KB: Data collection, analysis and interpretation, Critical revision of article, Statistics, Data collection. AH: Data interpretation, Drafting article, Critical revision of article, Approval of article. OS: Data interpretation, Drafting article, Critical revision of article, Approval of article, Statistics. RS: Drafting article, Critical revision of article, Approval of article. All authors read and approved the final manuscript.
